# Metformin Use and Vitamin B12 Deficiency in People with Type 2 Diabetes. What Are the Risk Factors? A Mini-systematic Review

**DOI:** 10.17925/EE.2024.20.2.7

**Published:** 2024-07-12

**Authors:** Michael Atkinson, Prashamsa Gharti, Thinzar Min

**Affiliations:** 1. Department of Diabetes and Endocrinology, Morriston Hospital, Swansea Bay University Health Board, Swansea, UK; 2. Diabetes Research Group, Swansea University Medical School, Swansea, UK; 3. Department of Diabetes and Endocrinology, Neath Port Talbot Hospital, Swansea Bay University Health Board, Swansea, UK

**Keywords:** Cobalamin, diabetes mellitus, metformin, vitamin B12, type 2 diabetes mellitus, vitamin b12 deficiency

## Abstract

**Background and Aim:** Metformin is recommended as the first-line agent for the management of type 2 diabetes following lifestyle and dietary changes. The long-term use of metformin has been associated with vitamin B12 deficiency. The aim of this review is to investigate the effect of metformin on vitamin B12 levels and identify any risk factors. **Method:** A literature search was conducted using MEDLINE, PubMed and ProQuest Central. Selected articles were peer-reviewed articles, written in English and published from 2015 and onwards. Excluded articles were case reports, reviews or meta-analyses, as well as those with no access to full text. **Results:** In total, 21 articles were included. There was a significant association between metformin use and vitamin B12 levels in 17 studies, while 4 studies found no such association. The risk factors examined were metformin dose, treatment duration, patient age and patient ethnicity. **Conclusion:** In summary, metformin use was associated with lower vitamin B12 concentrations, and higher doses and longer durations of treatment increase the risk of vitamin B12 deficiency. Routine vitamin B12 screening is recommended, prioritizing higher-risk patients. Further research is needed to identify when to initiate monitoring.

## Metformin

Metformin has been recommended as the first-l ine glucose-l owering agent for the management of type 2 diabetes (T2D) for several decades due to its efficacy and safety profile.^1–3^ In fact, metformin has been widely used as an insulin-sensitizing agent for not only T2D but also pre-diabetes and polycystic ovary syndrome. The common side effects of metformin are gastrointestinal symptoms. Rare side effects include lactic acidosis and vitamin B12 deficiency. The mechanisms of action of metformin include inhibiting hepatic gluconeogenesis, enhancing peripheral glucose uptake and utilization in the skeletal muscle, and delaying intestinal glucose absorption.

Metformin undergoes enteric absorption through plasma membrane monoamine transporters situated on enterocytes.^4^ It is then distributed from the circulation to its target sites, including the liver and skeletal muscles, through organic cation transporters 1 and 3. Due to the negative membrane potential of the inner hepatic mitochondrial membrane, and the positive charge on metformin, the drug accumulates in the mitochondria.^5,6^ Metformin inhibits complex I of the electron transport chain, which reduces adenosine triphosphate (ATP) production. As hepatic gluconeogenesis depends on sufficient ATP levels, this reduction in cellular energy disrupts glucose production. Low cellular energy levels also activate adenosine monophosphate kinase-activated protein kinase (AMPK). To restore normal energy levels, AMPK alters ATP production, inhibiting processes that consume ATP.^7^ This also stimulates the breakdown of nutrients, such as fatty acid (FA) oxidation, by phosphorylating acetyl-c oenzyme A (CoA) carboxylase to enable FA to accumulate in the mitochondria. This stimulation of FA oxidation in the liver reduces lipid levels and promotes insulin sensitivity.

## Vitamin B12 deficiency

Vitamin B12, also known as cobalamin, is a water-soluble vitamin, primarily obtained from animal-sourced foods, such as red meat, milk, eggs, poultry and shellfish. Vitamin B12 is essential for optimal neurological functions and the haematopoietic system.^8^ Vitamin B12 deficiency can have significant clinical consequences due to its important role as a cofactor for methylmalonyl-CoA mutase in the conversion of methylmalonic acid (MMA) into succinic acid and the conversion of homocysteine into methionine.^8,9^ Increased MMA and homocysteine levels can cause myelopathy and peripheral neuropathy. Vitamin B12 is also necessary for the synthesis of oligodendrocytes, which are myelin-producing nerve cells in the central nervous system.^10^ Disrupted myelin production caused by deficient vitamin B12 concentrations can result in poor regenerative ability of the myelin following injuries.

The prevalence of vitamin B12 deficiency was 6% in adults younger than 60 years and 20% in adults older than 60 years.^11^ The prevalence was higher in those with T2D, up to 30%.^12,13^ Vitamin B12 deficiency can be especially detrimental in people with T2D as the vitamin B12-related neuropathy can be misinterpreted as diabetic peripheral neuropathy, delaying diagnosis and timely treatment of vitamin B12 deficiency.

It has been suggested that the long-term use of metformin has been associated with vitamin B12 deficiency.^8^ The exact mechanism by which this effect occurs is not yet fully understood, and several mechanisms have been considered. Vitamin B12 undergoes enteric absorption in the ileum by binding to intrinsic factors secreted by parietal cells in the stomach.^8^ The resulting complex allows vitamin B12 to bind to cubilin receptors on the enterocytes through endocytosis. Metformin is theorized to impair the absorption of vitamin B12 via several mechanisms. Firstly, it has been suggested that metformin impairs the calcium-dependent absorption of the vitamin B12–intrinsic factor complex at the enteric cubilin receptors in the ileum, with or without impairment of the cubilin endocytic receptor.^8^ Emerging evidence suggests this as the most plausible mechanism, as the inhibitory effect has been noted to reverse following calcium administration.^14^ Metformin may also delay small bowel motility, and the resulting bacterial overgrowth may again inhibit the absorption of the vitamin B12–intrinsic factor complex. Alternatively, metformin may inhibit the secretion of intrinsic factors from parietal cells.^15^ In terms of hepatic involvement, it is postulated that alterations in bile acid function may cause reduced enterohepatic circulation of vitamin B12.^8,16^

The aim of this review is to investigate the association between metformin use and vitamin B12 deficiency in people with T2D and identify risk factors.

## Methods

This review was performed in accordance with the Preferred Reporting Items for Systematic Reviews and Meta-Analyses (PRISMA) statement.

### Search strategies

An initial scoping search was performed using Google Scholar. Abstracts were analysed to identify synonyms of keywords. Search terms used reflected concepts, such as the prevalence of vitamin B12 deficiency in metformin users, risk factors, clinical trial data supporting or refuting an association and any recommended changes to the current preventative strategies. Keywords such as ‘type 2 diabetes’ or ‘type 2 diabetes mellitus’, ‘metformin’, ‘vitamin B12’ or ‘cobalamin’ and ‘prevalence’, ‘association’ or ‘cause’ were used to search in the following databases: MEDLINE, PubMed (done on 24 February 2022) and ProQuest Central (done on 2 March 2022) (see Appendices Appendix 1: Search terms used).

### Eligibility criteria

Original research articles published from 2015 onwards in peer-reviewed journals, written in English, were included. Review, meta-analyses, case reports and *in vitro* and animal studies were excluded.

### Data extraction

The following data were extracted from each study: the first author and publication year, type of study, duration of study, number and characteristics of participants, duration of metformin use, dosage of metformin use and cut-off definition of vitamin B12 deficiency. These data were presented in an article matrix.

### Statistical analysis

Due to the heterogeneity of the study designs and samples in each article, a straightforward statistical analysis was not feasible. Therefore, a qualitative summary of each article was presented, and a narrative synthesis was done to describe the associated risk factors.

## Results

### Selection process

**Figure 1:**
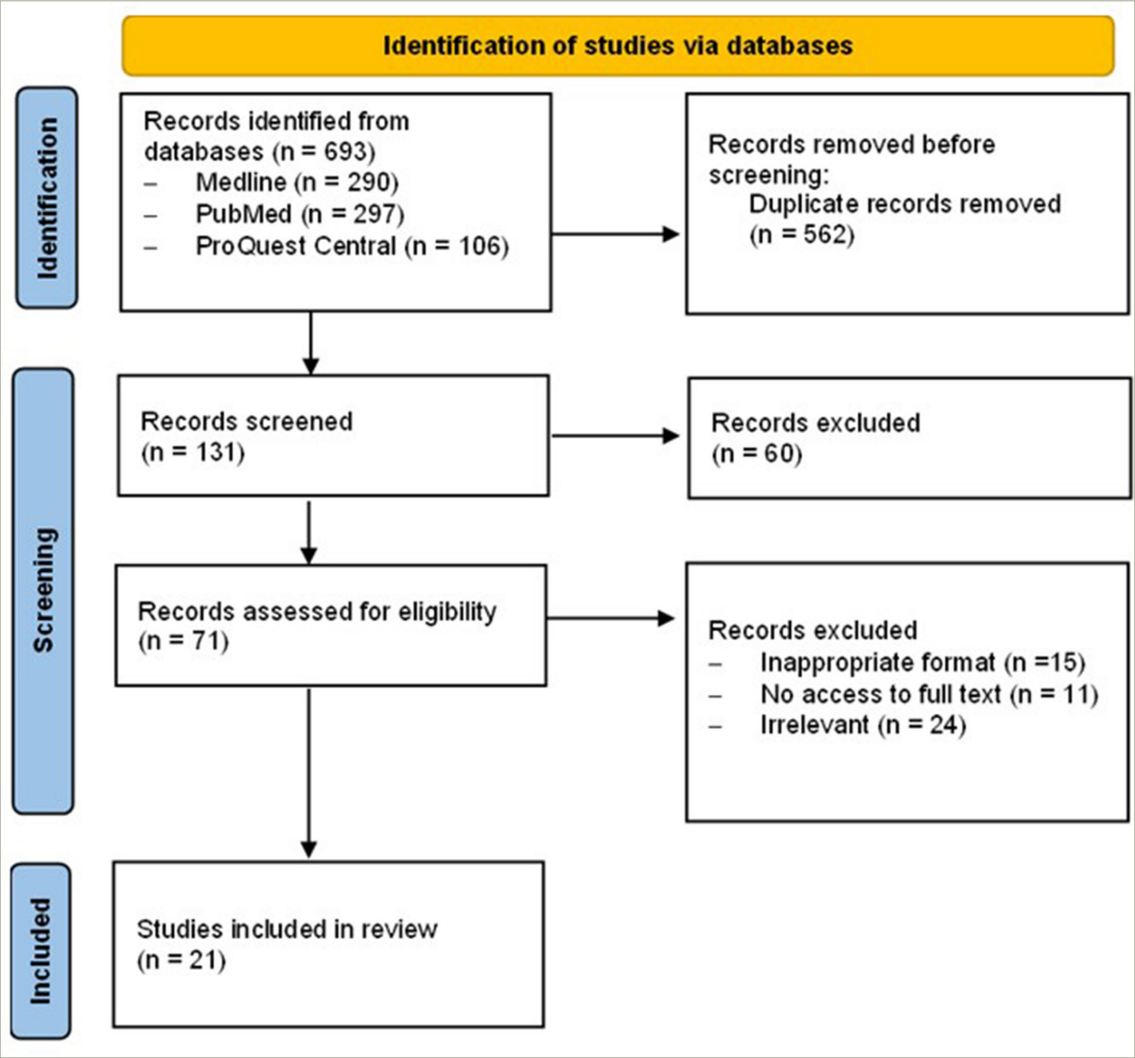
The Preferred Reporting Items for Systematic Reviews and Meta-Analyses (PRISMA) diagram

The literature search across three databases generated 639 articles. After the removal of 562 duplicates, 131 articles were screened, and 60 articles were excluded as per exclusion criteria. Overall, 71 articles were assessed for eligibility, and 21 articles were included in this mini-systematic review. The summary of the selection process is shown in the PRISMA flow diagram (Figure 1).^17^

### Study characteristics

The vast majority of the studies included were cross-sectional studies (12/21), with the remainder being case–control, observational or cohort studies and one randomized controlled trial. Studies included between 72 and 3,124 patients. The duration of diabetes was generally >2 years. Notably, Roy et al. studied patients with early diabetes of <5-year duration, and Kanti et al. included patients with the shortest duration of both diabetes and metformin therapy at 187–202 days and 170 days, respectively^18,19^. The duration of metformin use was sub-categorized in most studies. The shortest durations outlined were ≥3 months,^12,20^ ≥6 months^18^ and a mean of 187–202 days^19^ (as described in a subgroup study by Kanti et al.). Maximum and minimum doses of metformin were not reliably reported, but the range specified was from <500 mg up to >2,500 mg daily. There was some variation in the definition of vitamin B12 deficiency. Most studies selected <133–150 pmol/L as normal levels of vitamin B12. Outliers were reported by Kanti et al. at <35 pmol/L and those with a higher threshold of <200 (or <300) pmol/L (*Table 1*).^12,18–37^

### Supporting an association

Of the 21 studies, 17 supported a significant association between metformin use and vitamin B12 levels. Vitamin B12 levels were compared between metformin users and non-metformin users in 11 out of these 17 studies, while 6 studies looked at patients taking metformin without comparison with a control group. Alharbi et al. showed that vitamin B12 deficiency was more prevalent in the metformin group than in the control (9.4 versus 2.2%, p<0.036).^24^ This higher prevalence in metformin users is also supported by the data reported by Ali et al. (10.71 versus 3.21%, p=0.00), Miyan and Waris (3.9 versus 2.1%, p=0.002), Owhin et al. (41 versus 20%, p=0.001) and Shivaprasad et al. (59.1 versus 40.1%, p<0.001).^25,32,34,36^ An association was also quantified by significantly lower vitamin B12 levels in metformin users than non-metformin users, as shown by Hasan et al. (360 ± 185.2 versus 619 ± 176 pmol/L, p<0.0001), Kancherla et al. (409 versus 445 pmol/L, p=0.02) and Roy et al. (306.31 ± 176.70 versus 627.54 ± 168.32 mg/dL, p<0.001).^18,27,29^ Out et al. measured MMA as an indicator of low vitamin B12 levels and found that mean MMA levels were higher in metformin users (0.185 ± 0.073 to 0.222 ± 0.100 μmol/L) than in the control group (0.185 ± 0.081 to 0.200 ± 0.074 μmol/L).^33^

### Disproving an association

No association was found between metformin use and vitamin B12 deficiency in 4 out of the 21 studies. The prevalence of vitamin B12 deficiency was generally low in metformin users in these studies; only 3.6 and 8.8% of patients were deficient.^22,26^ No significant difference in vitamin B12 levels was observed between metformin and non-metformin users in the study by Elhadd et al. (331.24 versus 337.80 pmol/L, p=0.87), Kanti et al. (105.4 versus 97 pmol/L, p=0.31) and Sugawara et al. (521.8 ± 285.6 versus 518.4 ± 293.6 pg/mL, p=0.94).^19,26,37^

*This shows the selection process of the articles included, which were identified and screened based on eligibility^17^*

### Borderline deficiency

Miyan and Waris found that 27.9% of non-metformin users were borderline-deficient compared with 18.4% of metformin users (p=0.002).^32^ This was supported by Owhin et al. (80 versus 59%, p=0.001).^34^ Khan et al. showed that borderline deficiencies were also generally prevalent in patients with T2D, with 30.1% borderline deficient compared with 25.4% who were fully vitamin B12 deficient.^12^ Metformin users may have had an influence on this as 58.4% were on metformin treatment and only 27.8% were on a higher dose (>2,000 mg).

### Subgroup analyses of risk factors

#### Metformin dose

The effect of the dose of metformin on vitamin B12 levels was analysed in 10 studies, and 9 of these supported a significant correlation. Akinlade et al. compared vitamin B12 levels among metformin users on daily doses of greater or less than 1,000 mg and showed that those taking the higher doses had lower vitamin B12 levels (306.98 versus 417.29 pg/mL, p=0.004).^21^ Al-Hamdi et al. investigated vitamin B12 levels in metformin users taking 2,000 mg or more daily.^23^ Patients who were vitamin B12-deficient were taking a higher dose of metformin than patients with normal vitamin B12 levels (1,981 ± 222 versus 1,695 ± 494 mg, p=0.004), and 96.2% of patients who were deficient were prescribed 2,000 mg or more daily (p=0.004). A higher mean daily dose in vitamin B12-deficient patients was also found by Yousef Khan et al. (1,827.25 ± 400.35 versus 1,875.91 ± 323.46 mg, p=0.001) and Kim et al. (1,558 ± 438 versus 1,276 ± 472 mg/day, p<0.001).^30^ This correlation was supported by Alharbi et al., who reported that the risk of deficiency was significantly higher in those receiving a 2,000 mg daily dose compared with those receiving less than 1,000 mg (odds ratio [OR]=32.5 versus OR =4.12, p<0.01).^24^

Furthermore, other studies that did not find a significant association between metformin use and vitamin B12 levels did find a significant correlation between metformin dose and vitamin B12 levels.^20,22^ Compared with lower doses, patients taking a dose of 1,000 mg daily or more had a higher rate of borderline deficiency (71 versus 58%, p=0.023)^23^ and full deficiency (4.3 versus 2.5%, p=0.023).^22^ Sugawara et al. found a significant correlation with dose (correlation coefficient (*r*)=-0.33, p<0.01).^37^ Only one study did not support a dose-dependent effect, as the results were not statistically significant (>1,000 mg, OR =1.57 versus ≤1,000 mg, OR =1, p=0.33).^30^

**Table 1:** Article matrix: summary of key points from 21 selected articles

Studies selected	Type of study	Patient characteristics	Metformin Duration	Daily dose	Defined B12 levels	Conclusion
Akinlade etal.^21^	Cross-sectional study (n=81)	<10 years of metformin duration Average age: 60.76 ± 8.26 years>10 years of metformin durationAverage age: 60.76 ±8.26 years<1,000 mg daily metformin doseAverage age: 61.96 ±8.06 years>1,000 mg daily metformin doseAverage age: 61.96 ±8.78 years Sex and diabetes duration were not described	All >5 years <10 years (n-50) >10 years (n=31)	>1,000 mg (n=25) <1,000 mg (n=56)	Deficient: <200 pg/dL Borderline: 200-300 pg/dL Normal: >300 pg/dL	Metformin use associated with lower vitamin B12 levels, particularly in larger doses and longer duration
Al Saeed and Baraja^22^	Cross-sectional study (n=307)	Mean age (±SD): 58.6 ± 11.1 years Female: 62.2% Diabetes duration: <10 years: 55% >10 years: 45%93.2% non-smokers 99.7% no alcohol No vegetarians	Mean =9.53 ± 5.85 years	<1,000 mg >1,000 mg	Deficient: 150 pg/mL Borderline: 150-399 pg/mL Normal: >400 pg/mL	Low prevalence of vitamin B12 deficiency in metformin users Borderline deficiency prevalent in metformin users High-dose metformin associated with lower vitamin B12 levels
Al-Hamdi etal.^23^	Cross-sectional study (n=248) Three subgroups based on vitamin B12 levels: Deficient Borderline Normal	Mean age (±SD): 55.3 ± 10 years Female: 60.5% Diabetes duration: 6.5 ± 4.5 years	<4 years 4-10 years >10 years	>2,000 mg <2,000 mg	Deficient: <133 pmol/L Borderline: 133-200 pmol/L Normal: >200 pmol/L	Metformin use associated with lower vitamin B12 levels, particularly in larger doses Borderline deficiency prevalent in metformin users
Alharbi etal.^24^	Observational study (n=412) Categorized into two groups: Metformin users (n=319) Non-metformin users (n=93)	Metformin group: Average age: 57.8 ± 0.6 years Female: 53.9% Diabetes duration: 10.1 ± 0.7 yearsNon-metformin group:Average age: 56.6 ± 1.4 years Females: 74.31%Diabetes duration: 7.3 ± 0.8 years	<4 years >4 years	<1,000 mg 1,000-2000 mg >2,000 mg	Deficient: <132.8 pmol/L	Metformin use associated with lower vitamin B12 levels, particularly in larger doses and longer duration
Ali etal.^25^	Case-control study (n=280)	Metformin users (n=140) Age 37-60 years: 16.42% Age 61-90 years: 33.56%Female: 28.21% Males: 21,79%Diabetes duration: 32.11 ±8.66 monthsNon-metformin users (n=140)Age 37-60 years: 15.35% Age 61-90 years: 34.63% Female: 10.3% Male: 39.6% Diabetes duration: 30 ± 6.66 months	>1 year	Not specified	Deficient: <150 pmol/L	Metformin use associated with lower vitamin B12 levels
Elhadd etal.^26^	Observational study (n=362)	Metformin users Age: 54.19 ± 11.61 years Diabetes duration: 10.27 ± 7.45 yearsNon-metformin usersAge: 52.67 ± 13.95 years Diabetes duration: 12.89 ± 8.89 years Gender not specified	Not specified	Not specified	Deficient: <133 pmol/L	Low prevalence of vitamin B12 deficiency in metformin users No significant difference in vitamin B12 levels between metformin and non-metformin users
Hasan et al.^27^	Cross-sectional study (n=72)	Metformin users (n=40) Age: 56 ± 9.12 years Female: 55% Duration of diabetes: 7 ± 3.54 yearsNon-metformin users (n=32)Age : 60 ± 5.47 years Female: 35% Duration of diabetes: 8 ± 4.7 years	>2 years <2 years	Not specified	Deficient: <200 pmol/L	Metformin use associated with lower vitamin B12 levels
Hendrawati et al.^28^	Observational study (n=200) Categorized into two groups based on the duration of treatment: Group 1:1-3 years (n=100) Group II: >3 years (n=100)	Group I Female: 71% Age: <60 years: 53% >60 years: 47% Group IIFemale: 64% Age:<60 years: 36% > 60 years: 64%	Group 1:1-3 years Group II: >3 years	500-1,500 mg	Not specified	Metformin use associated with vitamin B12 deficiency symptoms, particularly in longer duration of treatment
Kancherla et al.^29^	Cohort study (n=2,510) Categorized into three groups: Metformin users (n=212) Non-metformin users (n=331) Patients without T2D (n=1,967)	Mean age (SD), years: Multivitamin use: 66.6 (8.8) No multivitamin use: 65.1 (8.7) Diabetes + metformin: 65 (8.2) Diabetes + no metformin: 67 (8.5) No diabetes/no metformin: 66 (8.8) Female: Multivitamin use: 66% No multivitamin use: 61% Diabetes + metformin: 59% Diabetes + no metformin: 60% No diabetes/no metformin: 65% Diabetes duration not specified	Not specified	Not specified	Deficient: <148 pmol/L Borderline: 148-221 pmol/L Normal: >221 pmol/L	Metformin use associated with lower vitamin B12 levels
Kanti etal.^19^	Cross-sectional study (n=249) Categorized into two groups: MET (n-123) NPT (n-126)	MET Age: 52.8 ± 11.6 years Female: 34% Diabetes duration: 187 ± 88 daysNPTAge: 54.4 ± 10.7 years Female: 39% Diabetes duration: 202 ± 91 days	170 ± 88 days	1,400 ± 600 mg	Deficient: <35 pmol/L	No significant difference in vitamin B12 levels between metformin and non-metformin users, noting the short duration of therapy (and short duration of diabetes)
Khan etal.^12^	Cross-sectional study (n=209)	Age: 66.49 ± 13.35 years Female: 45.5% Diabetes duration: 9.16 ± 5.59 years	>3 months	500-2,500mg	Deficient: <150 pg/mL Borderline: 150-350 pg/mL	Metformin use associated with lower vitamin B12 levels High prevalence of borderline deficiency in the T2D population
Kim etal.^30^	Cross-sectional study (n=1111)	Vitamin B12 deficiency Age: 60.1 ± 11.6 years Female: 40.1% Diabetes duration: 11.4 ± 7 yearsNormal vitamin B12Age: 59.3 ± 10.7 years Female: 42.5% Diabetes duration 10.2 ± 6.9 years	>6 months <10 years 10-20 years >20 years	<1,000 mg 1,000-1,500 mg 1,500-2,000 mg >2,000 mg	Deficient:<300 pg/mL	Metformin use associated with lower vitamin B12 levels, particularly in larger doses. No correlation between duration and vitamin B12 levels
Krishnan et al.^31^	Cross-sectional study (n=205)	Age (±SD): 56 (15.0) years Female: 61.5% Diabetes duration: 72 months No vegetarians Malay ethnicity: 78%	>5 years <5 years	>1,000 mg <1,000 mg	Deficient <300 pg/mL	Metformin use associated with lower vitamin B12 levels, particularly in longer duration
Miyan and Waris^33^	Prospective observational study (n=932) Categorized into two groups: Metformin users (n=645) Non-metformin users (n=287)	Metformin users Age: 51.16 ± 14.64 years Female: 56.6% Diabetes duration: 8.03 ± 5.4 yearsNon-metformin usersAge: 39.77 ± 14.95 years Female: 45.3%	Not specified	0-3,000 mg	Deficient: <200 pg/mL Borderline: 200-300 pg/mL Normal: >300 pg/mL	Metformin use associated with lower vitamin B12 levels Borderline deficiency prevalent in non-metformin users
Out etal.^33^	Randomized controlled trial Placebo (n=194) Metformin (n=196)	Metformin: Age: 64 ± 10 years Female: 59% Diabetes duration: 14 ± 9 yearsPlacebo:Age: 59 ± 11 years Female: 50% Diabetes duration: 12 ± 8 years	52 months	850 mg	Deficient: <150 pmol/L	Metformin use associated with increased MMA and, therefore, lowered vitamin B12 levels, particularly in larger cumulative dosages (dose and duration)
Owhin etal.^34^	Case-control study (n=200) Categorized into two groups: Metformin users (n=100) Non-metformin users (n=100)	Mean age: 55.8 ± 9.3 years Metformin group: Males: 49%Non-metformin group:Males: 51%Diabetes duration: Female: 5.14 ± 4.9 years Male : 4.92 ± 4.5 years	Not specified	Not specified	Deficient: <200 pg/mL Borderline: 200-399 pg/mL Normal: >400 pg/mL	Metformin use associated with lower vitamin B12 levels Borderline deficiency prevalent in non-metformin users
Raizada et al.^35^	Cross-sectional study (n=183) Categorized into two groups: Metformin users (n=121) Non-metformin users (n=63)	Metformin Age: 50.1 ± 11.5 years Female: 45.5% Duration of diabetes: 5.6 ± 4.71 yearsNon-metformin:Age: 49 ± 9.5 years Female: 51.7% Duration of diabetes: 1.99 ± 3.86 years	0-2 years 2-5 years >5 years	500-2,550 mg	Deficient <150 pmol/L Borderline 150-221 pmol/L	Metformin use associated with lower vitamin B12 levels
Royetal.^18^	Cross-sectional study (n=90) Categorized into three groups: Group A: Metformin with another OHA (n=20)Group B: Metformin monotherapy (n=35) Group C: Non-metformin users (n=35)	Group A Age: 49.45 ± 5.41 years Female: 50% Diabetes duration: <5 yearsGroup BAge: 50.46 ± 7.48 years Female: 71% Diabetes duration: <5 yearsGroup CAge: 50.46 ± 4.96 years Female: 60% Diabetes duration: <5 years	>6 months	S2g	Not specified	Metformin use associated with lower vitamin B12 levels
Shivaprasad et al.^36^	Observational study (n=2,887) Group 1: Metformin users (n=2061) Group II: Non-metformin users (n=826) MUI used to measure the metformin usage	Females: 33.9% Males: 66.1%Group l:Age: 49.1 ± 8.3 years Diabetes duration: 6.0 ± 3.8 yearsGroup IIAge: 48.5 ± 10.0 years Diabetes duration: 5.8 ± 3.6 years	5.0 ± 2.9 years	MUI = (dose (mg) x duration (year)) /1,000 patients categorized by MUI: 0 (Group II) <5(0.15-5) >5(5.1-10) >10(10.1-15)	Deficient <200 pg/mL Borderline 200-300 pg/mL Normal >300 pg/mL	Metformin use associated with lower vitamin B12 levels, particularly in higher MUI categories (larger doses and longer durations)
Sugawara et al.^37^	Cross-sectional study (n=185) Categorized into: Metformin users (n=122) Non-users (n=63)	Metformin user Age: 67.9 ± 7.6 years Female: 41% Diabetes duration: 16.7 ± 12.6 yearsControlAge: 70.4 ± 9.6 years Male: 68.3% Diabetes duration: 13.7 ± 13.9 years	0-15 years (mean, 6.6 ± 3.7 years)	0-2,000 mg (mean, 979.5 ± 491.2 mg)	Not specified	No significant association between metformin and vitamin B12 levels Correlation between metformin dose and vitamin B12 levels
Yousef Khan et al.^30^	Cross-sectional study (n=3,124)	Age: 56.59 ± 10.18 years Male: 71.57% Median duration of diabetes: 7 years (IQR 5-11)	>3 months	<1,000 mg 1,000-2,000 mg >2,000 mg	Deficient <145 pmol/L	Metformin use associated with lower vitamin B12 levels, particularly in larger doses No correlation between the duration of treatment and vitamin B12 levels

*IQR = interquartile range; MET = metformin; MMA = methylmalonic acid; MUI = Metformin Usage Index; NET = non-pharmacological treatment; OHA = Oral hypoglycaemic agent; SD = standard deviation; T2D = type 2 diabetes.*

#### Metformin duration

The effect of duration of metformin treatment on vitamin B12 levels was investigated in nine studies, and six supported a significant association. Vitamin B12 deficiency was more prevalent in patients who had been taking metformin for longer durations, with vitamin B12 deficiencies observed in 75% of metformin users of more than 4 years of treatment (p<0.001) compared with 6.25% in non-metformin users.^24^ Hendrawati et al. found a higher prevalence of neuropathy, a symptom of vitamin B12 deficiency, in longer metformin duration (69 versus 28%, p<0.001).^28^ However, it is likely that patients with a longer duration of metformin therapy also have a significantly longer duration of diabetes. It may be that the symptoms of neuropathy that are not specific to either aetiology are due to diabetic peripheral neuropathy rather than B12 deficiency in the absence of a confirmatory vitamin B12 level. The risk of deficiency was also increased in patients who had been taking metformin for more than 5 years (OR =2.27 versus 1, p=0.01).^31^ Akinlade et al. also support a correlation, with lower vitamin B12 levels found after 10 or more years of metformin usage (299.63 versus 429.48 pg/mL, p=0.004).^21^ Conversely, some studies showed no significant correlation between the duration of treatment and vitamin B12 levels (*r*=0.03, p=0.317; *r*=0.1, p=0.29; *r*=0.02, p= 0.10).^20,30,37^ It seems that the dose is a more influential factor as a significant correlation was only seen with the dose of metformin rather than with the duration of treatment (*r*=-0.30, p<0.001 versus *r*=0.03, p=0.317; *r*=-0.32, p=0.01 versus *r*=0.02, p=0.1).^20,30^

#### Age

*Table 2* shows a summary of the age of participants in all 21 selected studies. Thirteen out of the 21 articles investigated age as a confounding factor in vitamin B12 levels. Of these 13 articles, 8 supported a significant association between the two.^22,25–28,30,36^ Age differences were observed between patients in different metformin duration groups, with advancing age (≥60 years) associated with a longer duration of metformin treatment (group II 64% versus group I 47%, p<0.023).^28^ Hasan et al. demonstrated a higher average age of metformin users with deficient rather than normal vitamin B12 levels (61 ± 9.11 versus 54 ± 9.83 years old, p=0.042).^27^ This was also supported by Ali et al., who showed that vitamin B12 deficiencies were more prevalent in patients aged between 61 and 90 years compared with patients aged between 37 and 60 years (6.42 versus 1.42%, p=0.00).^25^ Furthermore, a negative correlation between age and vitamin B12 levels was demonstrated by Shivaprasad et al. (*r*=-0.14, p<0.001).^36^

**Table 2:** Age as a risk factor

Author/date	Eligible participants’ age (years)	Mean age (years)
Akinlade et al., 2015^21^	45–80	≤1,000 mg: 61.96 ± 8.06 >1,000 mg: 61.96 ± 8.78
Al Saeed and Baraja, 2021^22^	18–80	58.69 ± 11.1
Al-Hamdi et al., 2020^23^	≥18	55.3 ± 10.0
Alharbi et al., 2018^24^	≥18	MET users: 57.8 ± 0.6 Non-MET users: 56.6 ± 1.4
Ali et al., 2020^25^	40–80	MET users: 66.46 ± 10.39 Non-MET users: 67.38 ± 10.76
Elhadd et al., 2018^26^	N/A	MET users: 54.19 Non-MET users: 52.67
Hasan et al., 2019^27^	30–70	MET users: 59 ± 9.12 Non-MET users: 60 ± 5.47
Hendrawati et al., 2018^28^	N/A	60.18
Kancherla et al., 2016^29^	≥45	MET users: 65 Non-MET users: 67
Kanti et al., 2020^19^	18–69	MET: 52.8 ± 11.6 NPT: 54.4 ± 10.7
Khan et al., 2017^12^	≥45	66.49 ± 13.35
Kim et al., 2019^30^	20–85	59.5 ± 10.9
Krishnan et al., 2020^31^	≥18	56 ± 15.0
Miyan and Waris, 2020^32^	N/A	MET users: 51.16 ± 14.64 Non-MET users: 39.77 ± 14.95
Out et al., 2018^33^	30–80	MET: 63.6 ± 9.6 Placebo: 59.1 ± 11.0
Owhin et al. 2019^34^	≥30	MET users: 59.20 ± 8.92 Non-metformin users: 55.33 ± 9.19
Raizada et al., 2017^35^	>30	MET users: 50.1 ± 11.5 Non-MET users: 49.6 ± 9.5
Roy et al., 2016^18^	35–70	Group A: 49.45 ± 5.41 Group B: 50.46 ± 7.48 Group C: 50.46 ± 4.96
Shivaprasad et al., 2020^36^	≥20 and ≤65	Group I: 49.1 ± 8.3 Group II: 48.5 ± 10.0
Sugawara et al., 2020^37^	N/A	Control: 57% ≥70 Metformin users: 44% ≥70
Yousef Khan et al., 2021^20^	≥18	Normal VitB_12_ levels: 56.4 ± 10.13 Deficient VitB_12_ levels: 57.03 ± 10.28

*Summary of key information from the 21 selected papers given in Table 1, identifying the age requirements for eligible participants, and the mean patient age in each subcategory MET = metformin; N/A = not available; NPT = non-pharmacological treatment; VitB_12_ = vitamin B_12_.*

No significant difference in age between vitamin B12-deficient and normal vitamin B12 groups was demonstrated in 5 out of the 13 articles.^20,24,31,32,37^ Even when metformin users were older, no correlation was observed between age and vitamin B12 levels (*r*=0.172, p<0.0001).^32^ A subset analysis of elderly patients aged 70 years and above in the metformin and control groups showed no significant differences in vitamin B12 levels (541.1 ± 330.0 versus 550.1 ± 303.9 pg/mL, p=0.90).^37^ Other risk factors were more significant than age, for example, metformin dose (≥2,000 mg, OR = 8.67, p<0.001 versus OR =1.02, p=0.019) and duration (>5 years, OR =2.07, p=0.01 versus OR =1.03, p=0.06).^30,31^

#### Ethnicity

The following countries were represented in the 21 selected articles: Germany, India, Indonesia, Japan, Malaysia, the Netherlands, Nigeria, Oman, Pakistan, Qatar, Saudi Arabia, South Korea and the USA. Pakistan was the most represented country and was the data source for four of the articles. Only one article investigated ethnicity as a risk factor for vitamin B12 deficiency, where most patients were Malaysian (78%).^31^ Non-Malay ethnicity was a more significant risk factor than metformin duration of 5 or more years (OR = 3.96, p<0.001 versus OR =2.06, p=0.049).

## Discussion

### Key findings

#### Metformin effect

A significant association between metformin use and vitamin B12 levels was supported. The use of metformin in patients with T2D reduces vitamin B12 levels, increasing the prevalence of vitamin B12 deficiency. A dose-and duration-dependent effect was also seen, whereby an increase in metformin dose and duration were significantly correlated with a decrease in vitamin B12 levels. These conclusions were supported by an analysis of six randomized controlled trials, which showed significantly lower vitamin B12 levels in metformin users than patients who received other interventions (mean difference, -53.93 pmol/L).^38^ A larger mean difference was observed in higher doses (≥2,000 mg/day, -78.62 versus -37.99 pmol/L). Although borderline deficiencies were generally prevalent in the T2D population and non-metformin users, the initiation of metformin therapy reduced vitamin B12 levels further into the deficient category.

A few selected articles did not find any significant association between metformin use and vitamin B12 levels. This could be due to the heterogeneity of the patients recruited in each study, representing a wide range of countries, metformin doses and durations. Elhadd et al. showed no significant association; however, the researchers did not assess metformin dose and duration in the patients selected.^26^ Two articles that did not find a link between the duration of therapy and vitamin B12 deficiency involved patients who had relatively short average metformin exposure compared with others. Patients in the study by Sugawara et al. had a mean duration of 6.6 ± 3.7 years, and eligible patients in the study by Yousef Khan et al. were prescribed metformin for a minimum of 3 months.^20,37^

#### Confounding factors

Age may be a confounding factor, as older metformin users had an increased risk of vitamin B12 deficiency. However, it seems that other risk factors, such as metformin dose and duration, were more significant and demonstrated a higher risk of deficiency. Other research supports this high prevalence of vitamin B12 deficiency in older populations, ranging from 5% up to 40% in the elderly.^39^ A decrease in vitamin B12 levels is often seen in normal ageing, with some suggestion of impaired vitamin B12 absorption from food.^40^ The progressive reduction in the gastric mucosa function during ageing causes a depletion in gastric acid, which is essential for the absorption of vitamins from food. Therefore, deficiencies in older patients may be due to age rather than metformin use. However, metformin use can reduce already vulnerable or borderline vitamin B12 levels into the deficient range.

The heterogeneity of patient ethnicity and the countries of origin could be another confounding factor. No significant differences in vitamin B12 levels were seen between Japanese metformin users and the control group.^37^ However, the authors suggest that due to the high fish and shellfish intake in Japan, the population generally have a lower deficiency risk regardless of metformin use. Research has shown that fish diets increase serum vitamin B12 levels more than meat diets containing chicken and pork.^41^ As vitamin B12 is primarily acquired through animal-based foods, vegetarian diets will likely be deficient in vitamin B12.^8^ Therefore, patients from India, where there is a high prevalence of vegetarianism, are at a higher risk of vitamin B12 deficiency due to reduced dietary intake.^36^

### Recommended guideline changes

Currently, in the National Health Service, routine vitamin B12 screening is not offered to patients with T2D.^42^ Vitamin B12 levels are likely to be checked only if symptoms suggestive of vitamin B12 deficiency are observed.^43^ This is likely to lead to cases of vitamin B12 deficiency being missed in patients who are asymptomatic or who have only early non-specific symptoms.^44^ Furthermore, symptoms of vitamin B12 deficiency, such as fatigue, are generic and can be misattributed to other causes.^45^ The literature suggests that there is a need for at least increased monitoring if not undergoing any treatment in asymptomatic cases.^46^ In patients with asymptomatic deficiency and ongoing need for metformin treatment, there may be an additional argument to treat such cases to prevent the development of symptomatic vitamin B12 deficiency. There is strong evidence for routine screening to be offered to patients on metformin therapy, particularly prioritizing older patients on larger doses with longer durations of treatment, as well as for those following vitamin B12-deficient diets. A randomized controlled trial on metformin users (n=5131) found that 44.5% were referred for vitamin B12 monitoring.^47^ The patients referred had a higher prevalence of diabetes-related complications, such as ischaemic heart disease (16 versus 7%, p<0.05). Chi-square testing revealed that as age increased, the less likely it was that patients had been tested for vitamin B12 deficiency. Conceptually, prioritizing testing for older patients was supported as vitamin B12 supplements were used more frequently in patients aged 46 years and above (81.7%, p=0.007), which raises the question of whether routine screening should be prioritized for high-risk patients.^48^

Current treatment for vitamin B12 deficiency not related to diet involves injection of a 1 mg dose of intramuscular hydroxocobalamin every 2–3 months.^42,49^ Although Parry-Strong et al. showed that sublingual supplements may be more effective in improving vitamin B12 levels, the higher initial dose may result in higher serum vitamin B12 levels after 3 months despite low bioavailability.^50^ No recommended changes to treatment are suggested as both injections and oral supplements are effective for low vitamin B12 levels.^47,50^

### Limitations

#### Proton-pump inhibitors

Polypharmacy was a confounding variable that was not investigated. In one selected study, 45% of patients were using medications to reduce gastric acid, primarily proton-pump inhibitors (PPIs).^12^ PPIs are prescribed to reduce gastric acid released by parietal cells in the stomach, which is important for vitamin B12 absorption and so can also decrease vitamin B12 levels. This suggests that the concurrent use of metformin and PPIs could be associated with a higher risk of vitamin B12 deficiency. Marcuard et al.^51^ showed that even a short exposure to omeprazole (for 2 weeks), a PPI, in healthy participants caused a reduction in vitamin B12 absorption. However, Ting et al. compared the long-term use of PPIs between patients with metformin-related vitamin B12 deficiency and controls and did not find any increased risk of deficiency.^52^ It is unclear if the concurrent use of PPIs influenced the data described, which reduces the quality of the evidence presented.

#### Defined vitamin B12 thresholds

A limitation of this review was that each study selected used different definitions for deficient and borderline vitamin B12 levels, so a patient who was classified as deficient in one study may not be categorized as deficient in another. Kanti et al. defined deficiency as less than or equal to 35 pmol/L, which was significantly lower than that reported by Hasan et al., who defined it as less than 200 pmol/L.^19,27^ This may have been important, as Kanti et al. showed no significant association between metformin and vitamin B12 deficiencies.^19^ National Institute for Health and Care Excellence guidelines state that a serum vitamin B12 level of less than 148 pmol/L is sufficient to clinically diagnose vitamin B12 deficiency.^45^ However, only one selected study uses this threshold.^29^ This inconsistency with diagnosing vitamin B12 deficiency prevents the generalizability to a wider T2D population, reducing external validity.

### Conclusion

Metformin is the first-l ine drug used for the management of T2D. This systematic review has confirmed that there is a significant association between metformin use and vitamin B12 deficiency, demonstrating that metformin use increases the risk of vitamin B12 deficiency. Patients receiving longer durations of metformin therapy and higher metformin doses are more susceptible to developing a deficiency. Patient age and ethnicity can also influence the risk of deficiency due to low vitamin B12 levels caused by ageing and diet. Routine screening is recommended for high-risk patients to reduce preventable vitamin B12 deficiency cases.

Further investigations are needed to identify the correlation between metformin dose and duration and the highest risk of developing vitamin B12 deficiency to understand when and how routine screening should be initiated. These preventative strategies will help detect vitamin B12 deficiencies earlier, so patients can be treated before complications occur, leading to a poorer quality of life and an increased socioeconomic burden.

## Appendices

### Appendix 1: Search terms used

Search conducted in databases MEDLINE and PubMed on 24 February 2022 and ProQuest Central on 2 March 2022. The following search terms (1–4) were used alongside the root search term.

**Table:** Table

#	Search terms
Root:	(‘Metformin’ [MeSH] OR metformin) AND (‘Diabetes Mellitus, Type 2’ [Mesh] OR ‘type two diabet*’ OR ‘insulin resistan*’) AND (‘Vitamin B 12 Deficiency’ [Mesh] OR ‘vitamin B12 deficien*’ OR ‘cobalamin deficien*’ OR ‘megaloblastic anemi*’ OR ‘megaloblastic anaemi*’)
Search 1	AND (prevalen* OR epidemi* OR occurrence* OR frequenc* OR distribut* OR common)
Search 2	AND (link* OR associate* OR relationship* OR relate* OR corelat*)
Search 3	AND (mechanism* OR caus* OR etiolog* OR aetiolog* OR ‘Molecular Mechanisms of Pharmacological Action’[MeSH])
Search 4	AND (treat* OR prevent* OR therapeutic* OR ‘therapeutics’[MeSH] OR ‘preventative medicine’[MeSH])
